# Dry eye in LASIK patients

**DOI:** 10.1186/1756-0500-7-420

**Published:** 2014-07-03

**Authors:** Mitsuyoshi Azuma, Chiho Yabuta, Frederick W Fraunfelder, Thomas R Shearer

**Affiliations:** 1 Senju Laboratory of Ocular Sciences Senju Pharmaceutical Corporation Limited, Kobe, Japan; 2 Department of Ophthalmology Oregon Health & Science University, Portland, OR 97239, USA; 3 Department of Integrative Biosciences Oregon Health & Science University, Portland, OR 97239, USA

**Keywords:** Dry eye, Human, LASIK, Schirmer’s reflex tear flow, Dry eye medication

## Abstract

**Background:**

Increasing age is a known risk factor for developing dry eye. The specific aims of the present study were to determine the prevalence of dry eye syndrome (DES) and use of post-operative dry eye medications in a relatively young population presenting for LASIK surgery at an academic ophthalmology clinic.

**Findings:**

A retrospective, analysis of 948 de-identified patient charts (median age 39 years, not age stratified) was performed to extract pre-LASIK diagnoses and post-LASIK medication lists. Clinical evaluation for DES and the results of Schirmer’s reflex tear flow test were used to assign LASIK patients into Normal, Pre-dry eye (Pre-DES), and Dry Eye Syndrome (DES) groups; which were then compared for use of dry eye medications.

Based on pre-operative diagnoses, only 2% (CI: 1.3 – 3.1) of LASIK patients presented with overt DES. Unexpectantly, 25% (CI: 22.2 – 27.6) of LASIK patients labeled Pre-DES were not classified by the clinician as having overt DES, yet they showed poor reflex tear flow rates ≤ 5 mm before surgery, and frequently used post-operative lubricant dry eye medications.

**Conclusions:**

Although the number of patients with pre-existing eye conditions was unknown, a sizable portion of relatively young LASIK patients displays poor reflex tear flow without overt DES. Such patients could go on to develop more serious consequences of poor tear flow, such as corneal abrasion and erosion. More specific, dry eye medications may be needed for ideal treatment.

## Findings

### Background

Dry eye is a multifactorial disease of the tears and ocular surface characterized by ocular discomfort visual disturbances, and potential erosion of the cornea. The underlying physiologic mechanism is believed to be an escalating cycle of interaction between tear film instability and tear film hyperosmolarity [1]. Activation of this self-enhancing cycle can be caused by many factors including LASIK-induced anesthesia of the corneal-lacrimal gland reflex, aged-related decreased tear production, diabetes associated neuropathy and microvascular changes, meibomian gland dysfunction, systemic and topical medications (β-blockers and atropine-like drugs), autoimmune acinar damage in Sjögren’s syndrome, herpes/HIV infections, and allergies [1].

The prevalence of dry eye in the US, Australia, and Asia comprising a total of 73,899 patients from 8 epidemiologic studies, ranged from 8 to 34% [2]. The vast majority of these patients were older than 40 years of age. Increasing age is a known risk factor for developing dry eye [2]. Obviously, good clinical care would ameliorate dry eye before it leads to corneal abrasion or impacts quality of life. We hypothesized that a substantial number of patients receiving LASIK would need continuing care for dry eye. Therefore, the specific aims of the present study were to determine the prevalence of dry eye and the use of post-operative dry eye medications in the relatively young patients presenting for LASIK surgery at an academic ophthalmology clinic.

## Methods

De-identified data were retrieved from medical charts from patients presenting at the Casey Eye Institute at the Oregon Health & Science University, according to a protocol approved by the Oregon Health and Sciences University Institutional Review Board and conducted in compliance with the Declaration of Helsinki (2008). Median age was 39 years (range 18 – 72), 36% male and 43% female (21% unknown), 83.3% of patients were Caucasian, and the patients were not stratified by age. Since the medical charts were anonymized, no consent was possible or required. Searching for LASIK billing codes identified relevant medical charts. Then the diagnosis (before LASIK) and the most recent medications list after LASIK were extracted from each chart in one computer search. Depending on where the patient was in his recovery, the medication lists would thus be at different times points between different patients, usually within 90 days, and always after LASIK. Schirmer’s reflex tear flow was performed without anesthesia [3] before LASIK, using ≤ 5 mm, as an indication of poor reflex tear flow. One ophthalmologist, with knowledge of the Schirmer’s test results, diagnosed DES (FWF).

## Results

**Figure 1:**
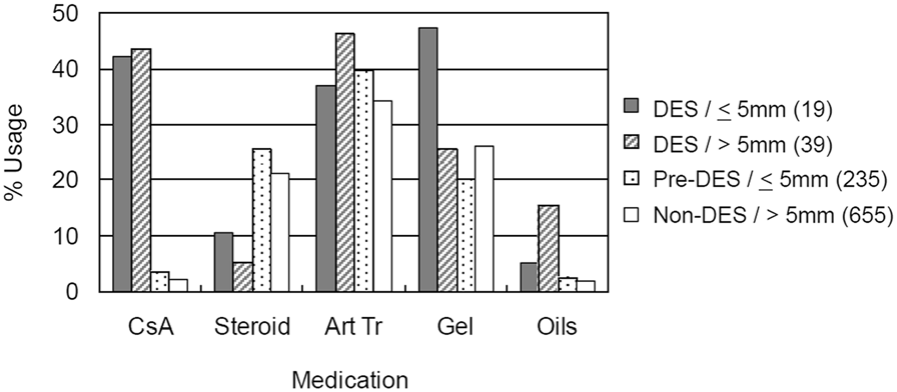
**Frequency of use of multiple dry eye medications after LASIK.** Numbers in () are total patients in each diagnostic group used to calculate the “% Usage” within each group. The group classification was based on the pre-operative diagnosis. Groups total > 100% because individual patients used more than one medication.

Of the total 948 patients undergoing pre-screening exams prior to LASIK surgery, only 2% had been diagnosed with DES and had low Schirmer’s reflex tear flow test values ≤ 5 mm (Table 1, first row). Another 4% were diagnosed with clinical DES but had adequate Schirmer’s reflex tear flow values (second row). The large number of patients (25%) not diagnosed with clinical DES but showing poor Schirmer’s reflex tear flow values (3rd row), were classified as susceptible to development of DES, and were labeled Pre-DES. Sixty-nine% of the patients presenting for LASIK surgery were “normal” in that they were clinically diagnosed as non-DES and had adequate Schirmer’s reflex tear flow values (4th row).After LASIK, the anti-inflammatory agent cyclosporine A (CsA) was used frequently by DES patients (Figure 1 grey and cross hatched bars), but not by the large group of Pre- DES patients (Figure 1, stippled bars). We also found that non-dry eye patients as well as DES patients used lubricant dry eye medications frequently. For example, artificial tears and gels use was very popular in both groups.

**Table 1:** LASIK patients grouped according to dry eye syndrome (DES) and Schirmer’s test values prior to surgery

**Diagnosis/Schirmer’s**	**Patients**	**%**	**95% CI**^**4**^
*DES*^*1*^*/≤5 mm*	*19*	*2*	*1.3 – 3.1*
*DES/>5 mm*	*39*	*4*	*3.0 – 5.6*
*Pre-DES*^*2*^*/≤5 mm*	*235*	*25*	*22.2 – 27.6*
Non-DES^3^/>5 mm	655	69	66.1 – 72.0
Total	948	100	

^1^DES was diagnosed in one or both eyes by a clinical ophthalmologist using: patient complaint, Schirmer’s reflex tear flow test, and fluorescein corneal surface staining (when appropriate).

^2^Pre-DES: ≤5 mm on Schirmer’s test in either eye and not diagnosed as DES.

^3^Non-DES: >5 mm on Schirmer’s test in either eye and not diagnosed as DES.

^4^95% confidence interval based on binomial distribution was calculated with JMP ver. 10.0.2 (SAS Institute, Cary, NC, USA).

## Discussion

The current study found that a cumulative total of 31% of patients in this relatively young group of patients presenting for LASIK surgery were affected to some degree by signs or symptoms associated with DES (Table 1, italic area).

This was especially surprising in the large group of patients labeled Pre-DES, who were not classified by the clinician as having overt DES, but who showed poor Schirmer’s reflex tear flow rates ≤ 5 mm. This is important because at least some of these patients would presumably go onto develop more serious consequences of poor tear flow such as corneal abrasion and erosion. A limitation of the current study was the number of patients with preexisting eye conditions was unknown.

The data also beg the question as to what preventive actions or medications should be administered to prevent these sequelae.

OTC artificial tears and gels were very popular in DES, Pre-DES, even in the Non-DES groups. The effectiveness of popular artificial tears used by pre-dry eye and dry eye LASIK patients will become limited if their symptoms progress. Other medications, having different mechanisms of action and possibly fewer side effects may be useful. These may include lacritin [4], PACAP (pituitary adenylate cyclase-activating peptide [5], and FK962 (N- (1-acetylpiperidin-4-yl)- 4-fluorobenzamide) [6].

## Conclusions

With the propensity of LASIK to exacerbate dry eye, more specific and/or customized medications for dry eye, such as those presented above, may be needed for the future.

### Consent

Anonymized data only; no consent needed.

## Abbreviations

CsA: Cyclosporine A, prescription anti-inflammatory agent inhibits lymphocyte activation; Ster: Steroids, prescription methylyprednisolone-receptor complex binds to DNA, to trigger anti-inflammatory and immunosuppressive responses; Art Tr: Artificial tears; OTC: Over-the-counter, buffered electrolyte solutions/viscosity agents; Gel: Gels OTC linked HMW polymers of acrylic acid to increase retention times; Oils: OTC mineral oils and petrolatum to increase tear film viscosity.

## Competing interests

Drs. Shearer and Fraunfelder are paid consultants for Senju Pharmaceutical Co., Ltd., a company that may have a commercial interest in the results of this research and technology. Drs. Azuma and Yabata are employees of Senju Pharmaceutical Co., Ltd. These potential conflicts of interest were reviewed, and a management plan approved by the OHSU Conflict of Interest in Research Committee was implemented.

## Authors’ contributions

MA and TRS conceived and designed the study, CY performed analysis of the data, FWF performed clinical evaluations, CY, MA and TRS drafted and revised the manuscript. All authors read and approved the final manuscript.
